# Beyond Difficult Discussions: Six Tools for Debriefing Tender Conversations in Healthcare

**DOI:** 10.12688/mep.20990.2

**Published:** 2025-05-16

**Authors:** Matthew Bowker, Amy Younger, Amy Huggin

**Affiliations:** 1 Newcastle University Faculty of Medical Sciences, Newcastle upon Tyne, England, UK; 2 Northumbria Healthcare NHS Foundation Trust, North Shields, England, UK

**Keywords:** simulation, debriefing, bad news, communication

## Abstract

**Background:**

Whilst debriefing literature offers valuable tools for healthcare education, there remains a gap in resources specifically designed for debriefing communication skills. Effective communication is fundamental to patient care, particularly during sensitive interactions. This article provides a specialised toolkit for educators to enhance communication skills debriefing, developed through synthesis of existing literature and the authors' extensive experience teaching communication skills through simulation.

**Methods:**

Drawing from literature and the authors' extensive experience teaching communication skills through simulation, we present six interconnected tools: leveraging cognitive dissonance, recognising micro-ruptures in rapport, mapping communication to clinical reasoning, differentiating sincere from performative empathy, metaphor dissection (analysis of the implicit meanings in patients' figurative language), and emotional labour accounting (the work of managing displayed emotions in professional contexts). We demonstrate these concepts through a fictional case study of Dr Morton's interactions with a patient and family.

**Results:**

The toolkit offers specific debriefing questions for each component that encourage reflective practice. Cognitive dissonance exploration helps clinicians recognise when competing professional values affect communication. Micro-rupture identification aids in preserving therapeutic relationships. Communication mapping enhances clinical decision-making. Understanding different forms of empathy guides appropriate engagement. Metaphor analysis reveals hidden meanings in patient-clinician dialogues. Emotional labour accounting acknowledges the personal cost of managing emotions professionally. Together, these elements create a framework that strengthens communication effectiveness whilst supporting clinician wellbeing.

**Conclusions:**

Effective debriefing of communication skills requires attention to both technical and emotional dimensions of healthcare interactions. This toolkit provides practical strategies for educators to help learners navigate the complexities of healthcare communication, ultimately improving patient care whilst supporting clinician resilience.

## Introduction

Debriefing literature largely focuses on a valuable set of skills and tools which can be widely applied. There is a relative dearth of literature on tools to support educators who are debriefing communication skills in particular. This article presents a set of tools and serves as an adjunct to traditional debriefing, shining a light on the art of communication and offering a space to refine these skills. The article is informed by literature and the authors’ substantial experience of teaching communication skills through simulation.

By debriefing our conversations, we open a window into the nuances of patient and family interactions. We explore not only what was said, but how it was conveyed, dissecting the layers of empathy, power, and emotional labour that underpin our dialogue. Through reflective discussion, we examine both verbal and non-verbal cues, recognising that every word and gesture carries meaning.

Our aim is to provide a practical toolkit that complements usual debriefing techniques, encouraging us to ask not just, “What went wrong or right?” but also, “How did our communication shape the experience?”. This toolkit is primarily designed for educators facilitating debriefing sessions following simulated communication scenarios in healthcare education settings. It can be implemented in both individual and group contexts, with particular value in structured simulation debriefing sessions where learners engage with standardised patients or role-play exercises. Secondarily, the toolkit offers value for peer-observation feedback discussions and self-reflection on recorded clinical interactions. By providing specific debriefing questions for each component, we aim to support educators in guiding learners through reflective practice that addresses both technical and emotional dimensions of healthcare communication. In doing so, we pave the way for more effective, compassionate communication that honours the humanity at the heart of healthcare.

## Tips

### A tender conversation

Whilst terms like "difficult discussions", "challenging conversations", or "courageous conversations" are commonly used to describe sensitive interactions, these labels can inadvertently create barriers by emphasising struggle. We propose that alongside these established descriptors, the concept of "tender conversations" offers a valuable complementary framework. As Mannix (2021) suggests, the word "tender" acknowledges both sensitivity and potential pain while avoiding defensive postures, helping clinicians lean towards empathy and care rather than steeling themselves for a challenge ^1^. This conceptual reframing does not replace established terminology but rather enriches our understanding of the emotional and relational dimensions of these interactions. We can translate theory into practice by breaking down real-world examples from our case study with Dr Morton. Although fictional, the examples from Dr Morton’s case are informed by the authors’ extensive experience teaching and debriefing communication skills. The goal is simple: to reveal how our language and non-verbal signals can inadvertently betray hidden biases, and to offer practical questions that help us refine our communication in future encounters.

### Case vignette

Dr Morton meets with the Thompsons in a quiet, private consultation room. Here, she is set to discuss a challenging update regarding their father, Arthur. As Mr and Mrs Thompson listen, subtle expressions of concern and hope are evident. In this setting, every word and gesture contribute to a dialogue where clinical clarity is balanced with compassionate engagement.

### A toolkit

***Cognitive dissonance***

In clinical practice, there are moments of tension between conflicting but sincerely held inner beliefs – a quiet inner tug-of-war called cognitive dissonance. Cognitive dissonance – first described by Festinger in 1957 – describes the internal discomfort experienced when holding conflicting beliefs ^2^. This internal tension, when left unexamined, can reveal hidden biases that seep into our language and gestures, subtly shaping the care we provide ^3^.

Consider this exchange:

*Dr Morton has noticed Arthur is becoming more short of breath and seems quite distressed. She asks him how he feels about his symptoms.*

**Arthur:**           “I’m worried about my symptoms getting worse. I’m just not sure how I’ll cope…”

**Dr Morton:**    “What’s worrying you the most?”

**Arthur:**           “I think it’s the breathing. I’m worried that I’ll die gasping for breath.”

**Dr Morton:**    “I appreciate why that is weighing heavy for you. It might be useful to discuss what we can do to help with breathlessness. We often use small doses of morphine to gently ease the feeling of being short of breath—

**Arthur:**           “I don’t want morphine.”

**Dr Morton:**    “What’s concerning you about morphine?”

**Arthur:**           “I want to have all my faculties about me. My remaining time with my family is so precious, and I want to be fully present for all of it.”

**Dr Morton:**    “We would aim to keep you fully *with it*, and just manage your symptoms—”

**Arthur:**           “I’m not having it.”

Dr Morton faces a challenge. She is deeply motivated to help with Arthur’s symptoms, and believes they are quite manageable. She feels upset that, without appropriate intervention, Arthur faces a steady deterioration of his breathing which could be profoundly distressing. However, she also recognises and respects Arthur’s agency and autonomy. She is therefore at a crossroads and must be attentive to the cognitive dissonance arising within her.

This dissonance manifests in competing professional values: her duty to relieve suffering versus her commitment to patient autonomy. The medical knowledge she carries – that morphine could effectively ease Arthur's respiratory distress – clashes with her ethical obligation to honour his informed refusal. Each time she attempts to redirect the conversation toward what she believes is best practice, she risks undermining the very relationship necessary for effective palliative care.

In this moment, acknowledging her discomfort rather than dismissing it becomes crucial. By recognising her internal conflict, Dr Morton can explore alternative approaches that respect Arthur's wishes while still addressing his fears. Perhaps there are non-pharmacological interventions for breathlessness, or different medications with effects more acceptable to Arthur. Maybe focusing on his desire for meaningful time with family could lead to discussions about advance care planning that would ease his anxiety about future breathlessness.

The resolution lies not in eliminating the cognitive dissonance, but in using it as a signal that deeper exploration is needed. By transparently addressing Arthur's concerns and working collaboratively toward solutions that align with his values, Dr Morton can transform this moment of tension into an opportunity for person-centred care. In doing so, she models the reflective practice essential to palliative care – where awareness of our own discomfort becomes a compass guiding us toward more authentic, respectful engagement with those we serve.

When debriefing with your participants, consider asking:

- **Supressing conflict:** “Is it right to suppress your discomfort here? Or should you disclose it?”
- **Explore Your Inner Conflict:** “How did your own internal conflict - this cognitive dissonance - manifest during the conversation?”

***“Micro-Ruptures” in rapport***

In the delicate dance of difficult conversations, even the smallest missteps can unsettle the fragile rapport between clinician and family. These “micro-ruptures” - those fleeting moments when trust begins to fray - can be pivotal ^4^. Recognising and addressing these cracks is essential for repairing the therapeutic alliance ^5^.

Consider the following exchange that demonstrates a micro-rupture:

**Dr. Morton:**    “I have the test results, and they confirm that your father's condition has deteriorated significantly. We need to discuss the next steps right away.”

Although factually accurate, this statement rushes through the emotional moment. The absence of a pause to acknowledge the family's distress, coupled with a swift transition to planning, represents a micro-rupture that risks leaving them feeling unseen and unsupported.

Now, contrast that with a reparative approach that addresses this rupture:

**Dr. Morton:** *(Noticing a family member's downcast eyes)* “I note that this news may potentially come as a shock to you- could you share a bit of what you're feeling?”

Here, the clinician pauses to address the subtle rupture, inviting the family to express their emotions and beginning the process of rebuilding trust.

Ruptures often manifest through subtle shifts in engagement - a change in posture, reduced eye contact, shorter verbal responses, or emotional withdrawal. Being alert to these signals requires deep presence and attention to both verbal and non-verbal cues. When we notice these subtle changes, acknowledging them directly but gently can help rebuild connection. This might mean slowing down, creating space for emotion, or explicitly checking whether we've understood correctly.

After such encounters, consider these debriefing questions to guide reflection:

For retrospective analysis:

- "What signals first alerted you that the connection might be fraying?"
- "When you noticed that pause, what did you feel was missing in the conversation?"

For anticipatory guidance:

- "When you sense a disconnect in future interactions, what might help you address it in the moment?"

These questions help practitioners develop their sensitivity to rupture signals while also exploring what might prevent us from responding to them. By examining both the recognition and response aspects, we can better maintain the delicate thread of connection that supports tender conversations.

***Mapping communication to clinical reasoning***

Effective communication is the bridge between clinical data and compassionate care. When we pay close attention to the emotional cues and gently guide our conversations, we open the door to a richer, more nuanced understanding of the patient’s situation, ultimately informing better clinical decisions ^4^.

Consider the following exchanges:

**Dr Morton:**    “Your father is nearing the end of his life. Could you remind me what symptoms led you to seek help?”

In this case, the clinician moves abruptly from delivering distressing news into a clinical inquiry. This approach can leave the family feeling overwhelmed and unheard, and it risks missing important contextual details about the patient’s experience.

**Dr Morton:**    “I know this is a lot to take in, and it must be incredibly hard right now. Before we go further, could you help me understand what changes you’ve noticed in his condition?”

Here, the clinician first acknowledges the emotional weight of the situation, creating a pause that allows the family to process their feelings. By inviting them to share their observations, Dr Morton not only gathers key clinical details but also integrates the patient’s narrative into her reasoning process. This thoughtful transition helps ensure that clinical decisions are informed by both hard data and the lived experience of the patient.

When debriefing, consider using these questions:

- **The necessity of communicating well:** “Is communicating well a ‘nice to have’ extra, or is it necessary to deliver effective care?”
- **The impact of communication on clinical reasoning:** “How might sensitive communication open up additional insights that inform your clinical reasoning?”

***Differentiating sincere empathy from performative empathy***

At first glance, the distinction between sincere empathy and performative empathy might appear straightforward, with sincerity viewed as inherently beneficial and performative empathy perceived negatively. However, the reality is far more nuanced. Empathy, whether sincere or performative, plays a complex role shaped by context, intention, and the experience level of the practitioner.

Performative empathy involves the deliberate use of prepared empathetic statements or gestures designed to convey understanding, even if the emotional depth behind them might be superficial. While such performative expressions can risk feeling scripted or insincere, they nevertheless serve an important function in clinical interactions, particularly in contexts where clinicians may feel caught off guard or emotionally flustered ^5^. For instance, a clinician encountering a colleague in a corridor who unexpectedly shares news of recent bereavement may rely on brief, performative empathy to appropriately acknowledge the situation without creating an expectation of deeper engagement that might be inappropriate or impractical at that moment.

Conversely, sincere empathy arises naturally and spontaneously from genuine emotional resonance with another's experience. It reflects an authentic emotional engagement that communicates profound respect and validation of another's feelings ^6^. Sincere empathy "involves a shift from observing how you seem on the outside, to asking about what it feels like to be you on the inside, wrapped in your skin with your set of experiences and background, and looking out at the world through your eyes ^7^." It's about recognising the unspoken emotions behind the words and responding in a way that validates those feelings. This kind of empathy requires self-awareness, a willingness to confront one's own vulnerabilities, and an openness to the narrative that the patient or family is sharing. Importantly, sincere empathy often invites further emotional disclosure, acting as a doorway to deeper conversations, which can be both therapeutically valuable and emotionally taxing.

Clinicians often navigate between these two modes depending on situational demands, their own emotional state, and their communication proficiency. Novices, consistent with the Dreyfus model of skill acquisition, often rely heavily on pre-prepared empathetic statements due to limited experience and confidence ^8, 9^. While many experienced clinicians may develop greater flexibility in modulating their empathetic expressions, this capability varies considerably among individuals and contexts ^5^. Research suggests that the relationship between clinical experience and empathetic skill is complex rather than linear, with factors beyond years of practice – including personality, training, and personal lives – significantly influencing empathy ^10, 11^. Yannamani and Gale ^10^ describe this change as “evolution” in empathy, reflecting the nuanced variation that occurs.

This adaptability highlights an important consideration: performative empathy, far from being intrinsically negative, can indeed be effective. It serves as a structured tool that clinicians can rely on, especially when confronted with unexpected emotional disclosures or personal uncertainty. In some instances, carefully chosen performative statements can promote trust and provide sufficient emotional support, without the necessity – or appropriateness – of delving deeper into emotional territory.

Yet, the key risk lies in over-reliance on performative empathy. When empathy is reduced to a scripted response, it risks coming across as detached or insincere. Excessive use of scripted responses, devoid of genuine emotional resonance, can undermine trust, leading to a sense of superficiality or even emotional alienation ^12^ or disgust ^13^. This form of performative empathy – sometimes characterised as a kind of tick-box exercise that, despite its outward appearance, can feel hollow – is often easily recognised by patients ^8^. Rather than opening up a dialogue, performative empathy may inadvertently close it off, leaving the patient or family feeling misunderstood or dismissed. Hence, finding the ‘goldilocks zone’ – the appropriate balance between too little and too much empathy – becomes essential. However, this zone is not universally fixed; rather, it varies with individual preferences, cultural backgrounds, and clinical context. Therefore, clinicians must become attune to cues indicating when to adjust their empathetic engagement.

Reflective debriefing provides clinicians an invaluable opportunity to evaluate their use of empathy critically. Consider using the following questions:

- **Insight into types of empathy:** "In that particular moment, was your empathy primarily performative or sincere?"
  - **Follow-up:** “Was this a deliberate choice?”
- **What kind of empathy the patient needs:** "How did your level of emotional engagement align with the patient's needs?"

Ultimately, understanding that both performative and sincere empathy hold valuable roles in clinical practice equips healthcare professionals with a versatile toolkit.

***Metaphor Dissection: When “Fighting” Cancer Undermines Hope***

Metaphors deeply influence how patients, families, and clinicians conceptualise illness, treatment, and recovery. Common metaphors such as "fighting cancer," "battling disease," or "the journey of recovery" permeate healthcare conversations, shaping not only understanding but also emotional responses and decision-making processes ^14, 15^. While metaphors can simplify complex medical concepts and foster a sense of unity and hope, they also carry the potential to inadvertently generate feelings of guilt, defeat, or inadequacy if the patient feels they're "losing the fight" or failing in their "journey" ^16^.

Consider the following dialogue:

**Arthur:**           “I feel like I’m losing this battle. Every day it's harder to fight.”

**Dr Morton:**    “Arthur, I hear you using the word 'battle.' It sounds exhausting. Can we talk a bit about what that metaphor means to you?”

Here, Dr Morton gently acknowledges Arthur's metaphor, inviting him to explore its deeper implications rather than simply accepting it at face value. This reflective approach allows Arthur to unpack the emotional burdens implicit within his chosen language, providing an opportunity to reshape the conversation towards comfort, support, and realistic expectations, rather than a binary win-or-lose scenario.

Alternatively, patients may choose metaphors that downplay their experiences:

**Patient:**       “The pain is just background noise.”

**Clinician:**    “I notice you're describing your pain as 'background noise.' Could we explore a little more about what that's like for you?”

Recognising such metaphors as indicators of emotional or physical understatement empowers clinicians to delve deeper, potentially uncovering hidden distress or unmet needs ^17^. Addressing metaphors explicitly can help validate the patient's lived experience and facilitate more accurate clinical assessments and responsive care.

Debriefing around metaphor usage encourages clinicians to pay closer attention to patients’ language, thus cultivating greater empathy and insight. Reflective questions to guide discussion may include:

- **Dissecting metaphor use:** “What emotions or assumptions might underlie the patient's chosen metaphor?”
- **Acknowledging metaphor use:** “How did acknowledging and exploring the patient's metaphor impact the conversation?”
- **Challenging metaphors:** “Were there metaphors you used or accepted without challenge that might have shaped the patient's perspective or expectations?”
  - **Follow-up:** “Do you think it would be right to challenge them?”

***Emotional Labour Accounting***

We use the term *emotional labour accounting* to describe the price we pay when we manage the emotions we display in professional contexts. Emotional labour, a concept introduced by Hochschild, refers to the work of managing how we display our emotions ^18^ to match the demands of the situation. For example, if a patient discloses a distressing event to a clinician, the clinician exerts work to maintain a professional demeanour while inwardly feeling deeply distressed by the disclosure. Sustained high levels of emotional labour are associated with burnout and poor mental wellbeing ^19, 20^. In teaching communication skills, we should ensure that our care and consideration extends to include the caregiver as well as the patient. Therefore, it is worthwhile to open a dialogue to discuss the *cost* of the emotional labour, so healthcare workers are better equipped to handle these challenging situations. When we openly acknowledge the effort it takes to carry our emotions, we invite others to do the same.

Consider these exchanges from our vignette:

Dr Morton pauses outside the consultation room. The disease has progressed despite treatment. She's running thirty minutes behind schedule, aware of the patients still waiting. Having treated Arthur for two years, she feels heartbroken that treatment is failing.
Yet when she enters the room, her demeanour is composed and attentive: "I am sorry you have had to wait so long to be seen today. I am with you now and you have my undivided attention. Is there anything you would like to discuss before we review your scan results together?"

It is worth noting that at some stages of the clinical communication process, allowing a glimpse of one's authentic self into these circumstances can add real, honest value to a patient/family member interaction ^21^. This is a skillset that does not come easily and needs to be practiced with discernment. It should be employed when it would benefit the patient if that experience or true emotion is shared, in a way that ensures the focus of the conversation doesn't shift entirely onto the clinician. In Dr Morton's case, she might choose to briefly acknowledge her own sadness about the disease progression if she judges this would validate Arthur’s experience without burdening him with her emotions.

Following such an encounter, consider these reflective debriefing questions that can be applied more generically:

- **The cost of emotions:** “What did it cost you to hold space for that interaction?”
- **The sum of the costs:** “How might the accumulation of such encounters throughout a clinical day impact your emotional reserves?”
- **Personal feelings:** “In what ways did your own emotional experience shape the dynamics of the conversation?”
- **Suppressing feelings:** “Is it right that Dr Morton suppressed her feelings?”
  - **Follow-up:** “Is it okay when we reveal our distress to our patients?”

By examining these dimensions of emotional labour, we can better prepare healthcare professionals to navigate the complex interplay between professional demands and authentic human connection. This understanding shapes how we teach communication skills, emphasising both patient care and clinician wellbeing.

## Discussion and conclusion

A summary of the skills is shown in Table 1.

**Table 1.** Summary Table of Debriefing Skills for Tender Conversations.

Skill	Description	Application	Key Debriefing Questions
**Leveraging Cognitive ** **Dissonance**	Recognising internal conflicts between competing professional values and using this awareness to improve care	When clinician values (e.g., symptom relief) clash with patient preferences (e.g., refusing medication)	• "How did your own internal conflict manifest during the conversation?" • "Is it right to suppress your discomfort here? Or should you disclose it?"
**Recognising Micro-** **Ruptures**	Identifying subtle breaks in rapport and trust during conversations and actively repairing them	When noticing changes in engagement, reduced eye contact, or emotional withdrawal from patients/ families	• "When you noticed that pause, what did you feel was missing?" • "What signals first alerted you that the connection might be fraying?" • "What stopped you from addressing it in the moment?"
**Mapping ** **Communication to ** **Clinical Reasoning**	Integrating emotional awareness with clinical assessment to enhance decision-making	When transitioning between delivering difficult news and gathering clinical information	• "How did acknowledging the family's emotional experience change the flow of the conversation?" • "How might sensitive communication open up additional insights that inform clinical reasoning?"
**Differentiating ** **Sincere from ** **Performative ** **Empathy**	Understanding when to use authentic emotional connection versus structured empathetic responses	When deciding how to express empathy based on context, experience level, and patient needs	• "Was your empathy primarily performative or sincere in that moment?" • "How did your level of emotional engagement align with the patient's needs?"
**Metaphor Dissection**	Examining the underlying messages and impacts of metaphors used in healthcare conversations	When patients use metaphors like "fighting cancer" or "background noise" for symptoms	• "What emotions or assumptions might underlie the patient's chosen metaphor?" • "How did exploring the patient's metaphor impact the conversation?" • "Were there metaphors you accepted without challenge?"
**Emotional Labour ** **Accounting**	Acknowledging the personal cost of managing emotions in professional interactions	When suppressing personal feelings to maintain a professional demeanour or sharing authentic emotion judiciously	• "What did it cost you to hold space for that interaction?" • "How might the accumulation of such encounters impact emotional reserves?" • "Is it right that emotions were suppressed? Is it appropriate to reveal distress to patients?"
**Framing Tender ** **Conversations**	Reframing "difficult discussions" as "tender conversations" to emphasise care rather than challenge	When approaching sensitive topics with patients and families	• "How did your framing of the conversation affect your approach?" • "Did the language you used create barriers or bridges?"

Effective debriefing of communication skills emerges from understanding the dynamic interplay among various conversational approaches, cognitive dissonance, micro-ruptures, metaphor usage, emotional labour, and differentiated empathy. These elements do not simply coexist but actively shape one another, forming a synergistic framework essential to nuanced healthcare communication.

The concept of 'tender conversations' – whilst not replacing established terminology such as 'difficult discussions' or 'challenging conversations' – offers a complementary perspective that encourages empathy and openness in clinician-patient interactions. This perspective potentially influences clinicians' capacity to recognise subtle disruptions in rapport (micro-ruptures) and address them proactively rather than reactively. When educators frame sensitive discussions through this lens during debriefing sessions, they may facilitate greater attentiveness to relational dynamics that might otherwise be overlooked in purely technical analyses of communication.

Similarly, cognitive dissonance, far from being an isolated experience, serves as an internal signal that often coincides with micro-ruptures. When clinicians detect internal tension between their professional duties and personal emotions, they become more sensitive to relational strains occurring in the conversation. Reflective engagement with cognitive dissonance thus enhances clinicians’ ability to respond to micro-ruptures effectively, preserving the integrity of therapeutic alliances.

Metaphor usage interacts profoundly with both empathy and emotional labour. When clinicians sensitively decode patient metaphors, they naturally deepen empathy, moving beyond superficial reassurance towards genuine understanding. This enhanced empathy, however, also heightens emotional labour, necessitating mindful balancing of emotional engagement to prevent clinician burnout. Thus, metaphor sensitivity not only shapes patient interaction but critically influences clinician self-awareness and emotional sustainability.

The interplay between sincere and performative empathy further illustrates the interconnectedness of the toolkit elements. Clinicians who master shifting between authentic emotional resonance and structured empathic expressions may better regulate their emotional labour, allowing them to remain emotionally present without becoming overwhelmed. This balance, informed by reflective debriefing practices, reinforces the clinician’s ability to maintain therapeutic rapport and address relational strains dynamically and effectively.

Together, these communication skills interweave continuously, each amplifying the effectiveness of the other. They create a holistic approach that strengthens clinician-patient interactions, improving patient care outcomes and clinician resilience simultaneously.

## Ethics and consent

Ethical approval and consent were not required

## Data Availability

No data are associated with this article.
