# Supplementary material for: Beyond Difficult Discussions: Six Tools for Debriefing Tender Conversations in Healthcare
Source: MedEdPublish (2016). 2025 May 16;15:12. Originally published 2025 Apr 4. [Version 2] doi: 10.12688/mep.20990.2 (PMC12416291; doi:10.12688/mep.20990.2)
Supplement: Peer review history

# Reviewer report: Reviewer response for version 2

**DOI:** 10.21956/mep.22567.r41838

**Published:** 2025-08-28

**Authors:** Nathan Oliver

**Affiliations:** 1 University of Canberra, Canberra, Australia

Thank you for the opportunity to review the resubmitted manuscript. 

 Whilst a point-by-point response to my feedback would have made things a little easier for me to follow in terms of your amendments, I enjoyed seeing the evolution of the piece on my read through and appreciate your notes highlighting the changes. 

 I am happy with your responses and look forward to seeing the manuscript indexed.

If evidence from practice is presented, are all the underlying source data available to ensure full reproducibility?

Not applicable

Is the topic of the practical tips discussed accurately in the context of the current literature

Yes

Are all factual statements correct and adequately supported by citations?

Partly

Are the conclusions drawn balanced and justified on the basis of the presented arguments?

Partly

Are arguments sufficiently supported by evidence from the published literature and/or the authors’ practice?

No

Reviewer Expertise:

Simulation, Debriefing, Simulation Design

I confirm that I have read this submission and believe that I have an appropriate level of expertise to confirm that it is of an acceptable scientific standard.

# Reviewer report: Reviewer response for version 2

**DOI:** 10.21956/mep.22567.r41935

**Published:** 2025-06-14

**Authors:** Vimala Govindaraju

**Affiliations:** 1 University Malaysia Sarawak, Kota Samarahan, Malaysia

The toolkit is a thoughtful, practice-oriented contribution to simulation‑based education. It correctly targets nuanced aspects of communication that traditional clinical debriefing can overlook. However, to move beyond a conceptual framework to a validated educational tool, future research must gather empirical data, define constructs rigorously, and test real-world impact. It presents a toolkit of six interlinked strategies designed for educators to facilitate post-conversation reflection and growth via simulation-based training. Built from literature review and the authors' experience, the toolkit is illustrated through a fictional scenario involving a tense doctor‑patient‑family dialogue.

If evidence from practice is presented, are all the underlying source data available to ensure full reproducibility?

Yes

Is the topic of the practical tips discussed accurately in the context of the current literature

Yes

Are all factual statements correct and adequately supported by citations?

Yes

Are the conclusions drawn balanced and justified on the basis of the presented arguments?

Yes

Are arguments sufficiently supported by evidence from the published literature and/or the authors’ practice?

Yes

Reviewer Expertise:

Health communication, Human communication, Interpersonal Communication.

I confirm that I have read this submission and believe that I have an appropriate level of expertise to confirm that it is of an acceptable scientific standard.

# Reviewer report: Reviewer response for version 2

**DOI:** 10.21956/mep.22567.r41837

**Published:** 2025-05-23

**Authors:** Paul Phrampus

**Affiliations:** 1 Univeristy of Pittsburgh, Pittsburgh, USA

The authors have done a fine job at addressing my concerns and incorporating recommended edits/changes. I think there is much more clarity associated.

If evidence from practice is presented, are all the underlying source data available to ensure full reproducibility?

Partly

Is the topic of the practical tips discussed accurately in the context of the current literature

Yes

Are all factual statements correct and adequately supported by citations?

Partly

Are the conclusions drawn balanced and justified on the basis of the presented arguments?

Yes

Are arguments sufficiently supported by evidence from the published literature and/or the authors’ practice?

Partly

Reviewer Expertise:

simulation; debriefing; communications

I confirm that I have read this submission and believe that I have an appropriate level of expertise to confirm that it is of an acceptable scientific standard.

# Reviewer report: Reviewer response for version 1

**DOI:** 10.21956/mep.22482.r41502

**Published:** 2025-05-16

**Authors:** Nathan Oliver

**Affiliations:** 1 University of Canberra, Canberra, Australia

Thank you for the opportunity to review your work. Really interesting read with a lovely tone and cadence. I have separated my feedback into an overall section and then some point-by-point comments. I hope you find them helpful.

**Overall**

- Overall, I found this a fascinating read. I like the way the authors write and the terms they have used for the various dynamics described. Really engaging approach in bringing in the case study vignette, it was novel and interesting. There is certainly a lot of value for the readership and the article will take the conversation forward on debriefing.

 

- I did feel lost at times, wondering if we were relating to the debrief as one of Dr Morgan after watching her conversations, or whether it was a simulation. The approach with the case study was used differently and to different degrees from tip to tip as well which became a little distracting for me.

 

- There were many claims within the manuscript which the authors appear to have taken for granted (secondary perhaps to their experience and command of the literature) which didn’t appear to be referenced. This should be addressed.

**Point-by-Point**

- "Debriefing literature largely focuses on a valuable set of skills and tools which can be widely applied. There is a relative dearth of literature on tools to support educators who are debriefing communication skills in particular”.
- *A big claim, can you provide more references?*

 

- “By debriefing our conversations, we open a window into the nuances of patient and family interactions. We explore not only what was said, but how it was conveyed, dissecting the layers of empathy, power, and emotional labour that underpin our dialogue. Through reflective discussion, we examine both verbal and non-verbal cues, recognising that every word and gesture carries meaning”.
- *Nothing to add, just flagging a lovely paragraph*

 

- “Our aim is to provide a practical toolkit that complements usual debriefing techniques…”
- *I’m not sure enough is done to define what I meant by ‘usual’*

 

- “The word "tender" acknowledges both sensitivity and potential pain while avoiding defensive postures, helping us lean towards empathy and care rather than steeling ourselves for a challenge”.
- *I’m not so sure. Tender might also communicate a conversation that is intimate (which is may or may not be), or perhaps focused on comfort, after the challenging word (for example, “the tender conversation by the nurse after the bad news was delivered by the medical team today”.*

 

- “The goal is simple: to reveal how our language and non-verbal signals can inadvertently betray hidden biases”
- *This is a big statement to introduce here without further development! Although I acknowledge you do lean into bias again within the vignette, it would be good to do some work here to prepare the reader.*

 

- Case Vignette.
- *I wonder if it might be a good approach to paint this like a simulation scenario, especially as you frame your later questions as ones you might ‘ask your participants’?*

 

- **Supressing conflict:** “Is it right to suppress your discomfort here? Or should you disclose it?”
- *What is this the goal of this question? Are we presuming the participants are aware and acknowledging the cognitive dissonance, and that they are experiencing discomfort? It is also a closed-ish framed question. You might need to walk the reader a little more into this one.*

 

- **Explore Your Inner Conflict:** “How did your own internal conflict - this cognitive dissonance - manifest during the conversation?” 
- *I like this question!*

 

- *The questions you pose here all assume the participants have recognised that the **micro-rupture** has occurred, it would be good as the reader to be with you as the author for the moment. The questions feel like they are coaching us to solve a problem we aren’t aware of as debriefers yet.  *

 

- “ **The necessity of communicating well:** “Is communicating well a ‘nice to have’ extra, or is it necessary to deliver effective care?”
- *This feels like a high-risk question that might come across as patronizing. It is also quite a closed one without an easy launchpad for more insight and conversation.*

 

- “At first glance, the distinction between sincere empathy and performative empathy might appear straightforward, with sincerity viewed as inherently beneficial and performative empathy perceived negatively. However, the reality is far more nuanced. Empathy, whether sincere or performative, plays a complex role shaped by context, intention, and the experience level of the practitioner.”
- References required

 

- “Novices, consistent with the Dreyfus model of skill acquisition, often rely heavily on pre-prepared empathetic statements due to limited experience and confidence ^8, 9^. More experienced clinicians, by contrast, are adept at modulating their empathy, seamlessly blending sincere and performative elements depending on what is most appropriate to the moment ^5^.”
- *Spicy paragraph – many might say that they’ve known novices to be extremely adept at communicating empathy and clinical experts who as devoid of it. I think you need to qualify the statement as there is a lot more going on than Dreyfuss’ clinical expertise in effectively communicating empathy.*

 

- *I’m interested that you didn’t use the vignette in the empathy discussion.*

 

- **Follow-up:** “Was this a deliberate choice?”
- *You’ve outlined how one can choose performative empathy. Do we have control over our sincere empathy?*

 

- *The emotional labour section is quite profound, and the questions are strong.*

 

- *I like the inclusion of the Table. I would like to see another column around debriefing examples of how to bring it into the discussion during the debrief. It feels like a small missing element that would really improve the usability of the toolkit for others. *

 

- Discussion and Conclusion 
- *You spend the majority of the space here discussing the interplay between the factors you introduce, but I wonder if another story is in how these very elements sit within the context of the debrief itself – and how not only are they a part of the toolbox in debriefing communication moments, but they are living and live artefacts occurring in our own debriefing conversations.*

Thank you for the opportunity to review your work, I think it has the makings of an important addition to the conversation on debriefing.

If evidence from practice is presented, are all the underlying source data available to ensure full reproducibility?

Not applicable

Is the topic of the practical tips discussed accurately in the context of the current literature

Yes

Are all factual statements correct and adequately supported by citations?

Partly

Are the conclusions drawn balanced and justified on the basis of the presented arguments?

Partly

Are arguments sufficiently supported by evidence from the published literature and/or the authors’ practice?

No

Reviewer Expertise:

Simulation, Debriefing, Simulation Design

I confirm that I have read this submission and believe that I have an appropriate level of expertise to confirm that it is of an acceptable scientific standard, however I have significant reservations, as outlined above.

# Reviewer report: Reviewer response for version 1

**DOI:** 10.21956/mep.22482.r41499

**Published:** 2025-05-06

**Authors:** Paul Phrampus

**Affiliations:** 1 Univeristy of Pittsburgh, Pittsburgh, USA

This manuscript presents a reflective toolkit designed to enhance the debriefing of healthcare communication. The integration of emotional labour, cognitive dissonance, metaphor analysis, and empathy evaluation into post-communication reflection is both innovative and pedagogically useful. The inclusion of practical debriefing questions and a fictional case (Dr. Morton) supports applicability across educational settings.

 However, while the conceptual contributions are strong, there are several points that require clarification or revision to strengthen the manuscript’s clarity, terminological precision, and practical utility.

**Major Comments**

 1. Terminological Shift from "Difficult" to "Tender" Conversations

 The authors propose replacing the widely accepted term “difficult conversations” with “tender conversations.” While conceptually interesting, this change introduces potential confusion:

 The term “tender” may imply distress, vulnerability, or the delivery of bad news. Not all challenging healthcare conversations involve these emotional elements.

 This shift may lead to unintended implications or misinterpretations in practice and training.

**Recommendation**: Unless empirical support is available for the benefits of this reframing, consider using “tender” as a supplementary descriptor rather than a replacement. Further discussion on the risks and implications of such a terminological shift is warranted. I would recommend rephrasing the title of the article.

 2. Intended Use and Audience of the Toolkit

 The manuscript lacks clarity, and sometimes seemed confusing to this reviewer on the intended application:

 Is the toolkit for individual self-reflection, educator-led debriefs, simulation feedback, or retrospective review of actual conversations?

 This ambiguity reduces the utility for educators attempting to adopt or implement these tools.

**Recommendation**: Add a clear statement early in the manuscript that specifies the primary and secondary use-cases for the toolkit, including possible formats of implementation (e.g., individual, group, simulated or real-world contexts).

 3. Interpretation of the Micro-Rupture Example (Page 4 of 9)

 The example exchange in this section risks misleading this reviewer reader. The example indicated to the "mindful approach" is better understood as a recovery from a ruptured moment, not a contrasting standalone approach.

**Recommendation**: Clarify that the second statement represents a reparative response to an identified micro-rupture. Reframing this section as a demonstration of rupture and recovery would improve interpretive accuracy.

 4. Assertion Regarding Experienced Clinicians and Empathy (Page 5 of 9)

 The manuscript states that experienced clinicians are adept at modulating empathy. This claim is too generalized and not clearly supported by the referenced article (Larson & Yao, 2005).

**Recommendation**: Revise this claim to reflect the variability in empathetic performance regardless of years of experience. Either support with stronger evidence or soften the assertion by framing it as a possibility rather than a norm.

**Minor Comments**

 1.  Clarity of Abstract and Introduction

 While the abstract summarizes the key components, it does not sufficiently highlight whether the toolkit is evidence-informed, expert-opinion-based, or tested in any formal educational settings. Consider adding this clarification.

 2.  Consistency in Terminology and Tone

 Phrases such as “emotional labour accounting” and “metaphor dissection” are unique and rich, but may benefit from brief definitions or more accessible phrasing for general readers unfamiliar with those frameworks.

 3.  Typographical/Stylistic Suggestions

 Page 4: Consider restructuring the bulleted questions for micro-ruptures to make clearer which are retrospective vs. anticipatory.

**Overall Recommendation**

 Revisions Required 

 This manuscript has high potential and introduces a novel, thoughtful contribution to communication skill debriefing. Addressing the issues around terminology clarity, application context, and overgeneralization will significantly strengthen its clarity, accessibility, and impact.

If evidence from practice is presented, are all the underlying source data available to ensure full reproducibility?

Partly

Is the topic of the practical tips discussed accurately in the context of the current literature

Yes

Are all factual statements correct and adequately supported by citations?

Partly

Are the conclusions drawn balanced and justified on the basis of the presented arguments?

Yes

Are arguments sufficiently supported by evidence from the published literature and/or the authors’ practice?

Partly

Reviewer Expertise:

simulation; debirefing; ommunications

I confirm that I have read this submission and believe that I have an appropriate level of expertise to state that I do not consider it to be of an acceptable scientific standard, for reasons outlined above.

# Response

**Published:** 2025-05-12

**Authors:** Matthew Bowker

**Affiliations:** Newcastle University, Newcastle upon Tyne, England, UK

Dear Professor Phrampus, We warmly thank you for your thoughtful and constructive review of our manuscript. Your detailed feedback has significantly strengthened our work, and we have addressed all points in our revision. Your comment regarding empathy in experienced clinicians particularly provoked reflection among our team; thank you for challenging us to reconsider this assertion with more nuance.

**Major comments**

**1. Terminological Shift from "Difficult" to "Tender" Conversations**

 We agree that replacing established terminology without empirical support could create confusion. We have revised our approach to position "tender conversations" as a complementary framework rather than a replacement, and modified both our title and the relevant sections to reflect this balanced perspective. We now present the concept as an additional lens that can enrich understanding while maintaining connection to established terminology.

**2. Intended Use and Audience of the Toolkit**

 We appreciate this important observation about application clarity. We have added an explicit statement early in the introduction specifying that the toolkit is primarily designed for educators facilitating simulation debriefing sessions, with secondary applications for peer observation feedback and self-reflection on recorded clinical interactions. We've also clarified implementation formats.

**3. Interpretation of the Micro-Rupture Example**

 Thank you for highlighting this potential misinterpretation. We have reframed this section to clearly demonstrate the rupture-repair sequence, explicitly stating that the second statement represents a reparative response to an identified micro-rupture rather than a contrasting standalone approach.

**4. Assertion Regarding Experienced Clinicians and Empathy**

 We are grateful for this input. We have revised our claim to acknowledge the complex, non-linear relationship between clinical experience and empathetic skill, noting that factors beyond years of practice - including personality, training, and institutional culture - significantly influence empathy.

**Minor comments** We have addressed all minor comments by:

 (1) clarifying in the abstract that our toolkit was developed through literature synthesis and the authors' simulation education experience;

 (2) providing brief definitions for specialized terms upon first mention; and

 (3) restructuring the debriefing questions to distinguish between retrospective analysis and anticipatory guidance.

 Thank you again for your valuable contribution to improving our manuscript. 

 Warmest regards,

 Dr Matt Bowker on behalf of all authors
